# Changes in diet quality and body weight over 10 years: the Multiethnic Cohort Study

**DOI:** 10.1017/S000711452100012X

**Published:** 2021-11-14

**Authors:** Minji Kang, Carol J. Boushey, Yurii B. Shvetsov, Veronica W. Setiawan, Hee-Young Paik, Lynne R. Wilkens, Loic Le Marchand, Song-Yi Park

**Affiliations:** 1 Cancer Epidemiology Program, University of Hawaii Cancer Center, Honolulu, HI 96813, USA; 2 BK21 FOUR Education and Research Team for Sustainable Food & Nutrition, Department of Food and Nutrition, College of Human Ecology, Seoul National University, Seoul, Republic of Korea; 3 Department of Preventive Medicine, Keck School of Medicine and Norris Comprehensive Cancer Center, University of Southern California, Los Angeles, CA, USA; 4 Department of Food and Nutrition, College of Human Ecology, Seoul National University, Seoul, Republic of Korea; 5 Center for Gendered Innovations in Science and Technology Researches (GISTeR), Korea Federation of Women’s Science & Technology Associations, Seoul, Republic of Korea

**Keywords:** Diet quality change, Body weight change, Multiethnic populations, Cohort studies, Dietary patterns

## Abstract

High-quality diets have been found to be beneficial in preventing long-term weight gain. However, concurrent changes in diet quality and body weight over time have rarely been reported. We examined the association between 10-year changes in diet quality and body weight in the Multiethnic Cohort Study. Analyses included 53 977 African Americans, Native Hawaiians, Japanese Americans, Latinos and Whites, who completed both baseline (1993–1996, 45–69 years) and 10-year follow-up (2003–2008) surveys including a FFQ and had no history of heart disease or cancer. Using multivariable regression, weight changes were regressed on changes in four diet quality indexes, Healthy Eating Index-2015, Alternative Healthy Eating Index-2010, alternate Mediterranean Diet and Dietary Approaches to Stop Hypertension scores. Mean weight change over 10 years was 1·2 (sd 6·8) kg in men and 1·5 (sd 7·2) kg in women. Compared with stable diet quality (< 0·5 sd change), the greatest increase (≥ 1 sd increase) in the diet scores was associated with less weight gain (by 0·55–1·17 kg in men and 0·62–1·31 kg in women). Smaller weight gain with improvement in diet quality was found in most subgroups by race/ethnicity, baseline age and baseline BMI. The inverse association was stronger in younger age and higher BMI groups. Ten-year improvement in diet quality was associated with a smaller weight gain, which varied by race/ethnicity and baseline age and BMI. Our findings suggest that maintaining a high-quality diet and improving diet quality over time may prevent excessive weight gain.

Dietary recommendations, such as the Dietary Guidelines for Americans, emphasise the importance of following a healthy eating pattern across the lifespan to achieve and maintain a healthy body weight and to reduce the risk of chronic disease^(1)^. Several diet quality indexes (DQI) including the Healthy Eating Index (HEI), the Alternative Healthy Eating Index (AHEI), the alternate Mediterranean Diet (aMED) and the Dietary Approaches to Stop Hypertension (DASH) scores have been developed to assess overall dietary patterns^(1–5)^. Although there are differences in components and scoring system in each index, the concepts of a high-quality diet emphasise inclusion of a variety of vegetables, fruits, whole grains, fat-free or low-fat dairy products, seafood, lean meats and poultry, eggs, beans and peas, and nuts and seeds; and moderation of saturated fats and *trans*-fats, added sugars and Na^(1–5)^.

Several prospective studies have reported that dietary patterns consistent with dietary recommendations are beneficial to prevent long-term weight gain^(6–9)^. While the studies have investigated the relationship between baseline diet quality and subsequent weight change, few studies have examined concurrent changes in diet quality and body weight over time. A large US cohort study found that increases in AHEI-2010, aMED and DASH scores were associated with less weight gain over 20 years^(10)^. However, the participants in the study were mostly white individuals.

The Multiethnic Cohort Study (MEC), consisting of participants primarily of five race/ethnicities, African American, Native Hawaiian, Japanese American, Latino and White, has collected information on eating habits and body weight at baseline and 10-year follow-up surveys. Using the data from the MEC, we examined the associations of changes in diet quality assessed by four DQI (HEI-2015, AHEI-2010, aMED and DASH) with body weight change over 10 years, and whether the associations varied by race/ethnicity, age and BMI at baseline.

## Methods

### Study participants

The MEC is a prospective cohort established to investigate lifestyle and genetic factors and chronic diseases. In 1993–1996, the cohort enrolled more than 215 000 men and women aged 45–75 years, who were primarily of five race/ethnicities: African American, Native Hawaiian, Japanese American, Latino and White^(11)^. At cohort entry, participants completed a self-administered comprehensive questionnaire that included a quantitative FFQ (QFFQ). In a 10-year follow-up survey (2003–2008), 98 214 participants (45 %) repeated the questionnaire. Participants who completed the follow-up survey tended, at cohort entry, to be younger (58·3 *v*. 60·3 years), Japanese American (31 *v*. 23 %), Whites (27 *v*. 19 %), never smokers (48 *v*. 45 %), more educated (graduated college 34 *v*. 21 %), less obese (17 *v*. 21 %) and to have higher diet quality (HEI-2015 67·6 *v*. 66·4) compared with non-respondents; the proportion of female was similar (56 *v*. 57 %). The institutional review boards at the University of Hawaii and the University of Southern California approved the study. For the present study, we excluded participants who did not self-report as one of the five racial/ethnic groups (*n* 5246), who reported implausible diets based on total energy intake or its components at either survey (*n* 5930), who had a history of cancer or heart disease at either survey (*n* 23 783) or who had missing or invalid (<15 or >50 kg/m^2^) BMI at either survey (*n* 3137). Since health implications of body weight and weight change are different for individuals who are elderly or underweight, we restricted the analysis to participants younger than 70 years with normal or elevated BMI (≥18·5 kg/m^2^) at baseline. The final sample included 53 977 participants (23 521 men and 30 456 women).

### Dietary assessment and calculation of dietary indexes

At baseline and 10-year follow-up surveys, dietary data were collected using a QFFQ, which can be accessed at https://www.uhcancercenter.org/mec. The baseline QFFQ with > 180 food items was developed based on 3-d measured food records^(11)^. For each ethnic group, the contribution of each food item to the total intake of major nutrients of interest (e.g., fat, dietary fibre, vitamin A, carotenoids and vitamin C) was calculated. A minimum set of food items that accounted for at least 85 % of the nutrients was identified for each ethnic group and included in the QFFQ. Therefore, the final questionnaire list accounted for much more than 85 % of the intake of the major nutrients^(11)^. A calibration study showed satisfactory correlations (0·55–0·74) between the QFFQ and three 24-h dietary recalls, for each racial/ethnic group, especially after energy adjustment^(12)^. For the 10-year follow-up survey, the QFFQ was updated with modest changes in the design, food lists and examples given for each food item. Daily nutrient and food group intakes were calculated using the MEC food composition tables^(13,14)^.

Diet quality was assessed by four DQI (HEI-2015, AHEI-2010, aMED and DASH scores), which were calculated for the MEC as part of the Dietary Patterns Methods Project^(5)^. In brief, the HEI-2015 is a measure of diet quality to assess conformance of dietary intake with the Dietary Guidelines for Americans 2015–2020^(15)^. The maximum HEI-2015 score is 100 points and higher scores indicate closer adherence with the dietary guidelines^(15)^. There are nine adequacy components including total fruits, whole fruits, total vegetables, greens and beans, whole grains, dairy products, total protein foods, seafood and plant proteins, and fatty acids; and four moderation components including refined grains, Na, added sugars and saturated fat^(15)^. The AHEI-2010 is based on foods and nutrients predictive of chronic disease risk^(2,16–18)^. The maximum AHEI-2010 score is 110 points and comprised of eleven components which are scored 0 (worst) to 10 (best)^(2,16–18)^). The components are vegetables, fruit, whole grains, sugar-sweetened beverages and fruit juice, nuts and legumes, red/processed meat, *trans*-fat, long-chain (*n*-3) fats (EPA + DHA), PUFA, Na and alcohol^(2,16–18)^. The aMED is a modification index from the Mediterranean diet scale of Trichopoulou *et al.*^(4,19)^. The possible scores on aMED range from 0 to 9, with nine components scored 0 (worst) or 1 (best): vegetables, legumes, fruit, nuts, whole grains, red and processed meats, fish, ratio of monounsaturated to saturated fat and ethanol^(4)^. The DASH score is based on food and nutrients for hypertension management^(3,20)^. The overall DASH score ranges from 8 to 40 including eight components, scored 1 (worst) to 5 (best): fruits, vegetables, nuts and legumes, whole grains, low-fat dairy products, Na, red and processed meats, and sweetened beverages^(3,20)^.

### Body weight assessment

BMI (as weight in kg divided by height in m^2^) at baseline was computed from self-reported weight and height from the baseline questionnaire. BMI at the 10-year follow-up was calculated from weight reported on the 10-year follow-up questionnaire and height from the baseline questionnaire. Weight change between the two surveys was calculated by subtracting weight at baseline from weight at 10-year follow-up.

### Statistical analysis

Selected characteristics of study participants are presented as mean values and standard deviations for quantitative variables and percentages for categorical variables for men and women separately. Change of diet quality over 10 years was analysed with two approaches. In the first approach, DQI change between two surveys was calculated by subtracting diet quality score at baseline from diet quality score at 10-year follow-up. Since the scales for the indexes are varied, DQI changes were examined based on 1 sd of change in each index over 10 years (online Supplementary Table S1). In this approach, DQI changes were treated either continuously, per 1 sd increment, or categorically: greatest decline (≥ 1 sd decrease), moderate decline (0·5– < 1 sd decrease), stable (< 0·5 sd change), moderate increase (0·5– < 1 sd increase) and greatest increase (≥ 1 sd increase), with the stable group as a reference. In the second approach, DQI changes were categorised into four trajectories based on the median scores of each index at baseline as follows: (1) consistently low, below the median value at both surveys (reference), (2) high to low, from above to below the median, (3) low to high, from below to above the median and (4) consistently high, above the median value at both surveys.

Using multivariable linear regression, body weight changes (means and 95 % CI) were regressed on DQI changes (per 1 sd increment, by change categories or trajectories) in men and women separately. The following covariates were included in the models: race/ethnicity (African American, Native Hawaiian, Japanese American, Latino, White); at baseline, age (years), BMI (18·5 to <25, 25 to <30, 30 to <35, ≥35 kg/m^2^), education (≤high school, vocational school/some college, ≥college graduate), marital status (married, separated/divorced, widowed, never married), smoking status (never, former, current), multivitamin use (yes/no), diet quality score and, for women only, menopausal hormone therapy use (yes/no); at baseline and change between the two surveys, physical activity (hours spent in moderate or vigorous activity per d) and energy intake (kcal/d). For HEI-2015 and DASH, the models were further adjusted for alcohol intake (g/d) at baseline and change between the two surveys. Unlike in the first approach with adjustment for baseline diet quality scores, for trajectories of DQI changes in the second approach, baseline diet quality scores were not adjusted for since they were already built into the trajectory definition. We also conducted subgroup analyses by race/ethnicity, baseline age and BMI. Data were analysed using SAS software (version 9.4, SAS Institute, Inc.) and statistical significance was considered at *P* < 0·05.

## Results

The mean age at baseline was 55·6 (sd 7·1) years in men and 55·8 (sd 7·1) years in women (Table 1). Over 10 years, mean body weight increased by 1·2 (sd 6·8) kg for men (80·3 kg at baseline to 81·5 kg at 10-year follow-up) and 1·5 (sd 7·2) kg for women (66·7–68·2 kg) (online Supplementary Table S1). In general, DQI slightly improved over 10 years both in men (HEI-2015 score: 65·2–68·8 points) and women (69·0–72·3 points) and across the racial/ethnic groups (online Supplementary Table S1). Men and women with greatest increase in HEI-2015 score over 10 years were more likely to be younger, Japanese American, more highly educated and married, to have lower body weight, and were less likely to be African American or to use multivitamins at baseline, compared with those with stable HEI-2015 (Table 1), see online Supplementary Tables S2–S4 for baseline characteristics by change in AHEI-2010, aMED and DASH.

**Table 1.** Baseline characteristics by change in Healthy Eating Index-2015 over 10 years in the Multiethnic Cohort Study (Numbers of participants; mean values and standard deviations; percentages)

Characteristics	All	Change in Healthy Eating Index-2015*				
		Greatest decline	Moderate decline	Stable	Moderate increase	Greatest increase
Men (*n*)	23 521	1838	2452	9033	4320	5878
Age (years)						
Mean	55·6	56·9	56·2	55·9	55·3	54·7
sd	7·1	7·3	7·2	7·1	7·0	6·7
Race/ethnicity (%)						
African American	7·0	10·6	8·6	7·2	6·4	5·5
Native Hawaiian	7·9	7·4	8·6	7·4	7·5	8·8
Japanese American	35·1	23·3	27·2	32·9	37·8	43·6
Latino	20·7	23·3	22·0	21·5	19·9	18·6
White	29·2	35·4	33·6	30·9	28·4	23·4
Body weight (kg)						
Mean	80·3	81·8	81·4	80·4	79·8	79·6
sd	14·1	14·7	14·2	14·0	14·0	14·1
BMI (%)						
18·5 to <25 kg/m^2^	35·9	33·3	35·0	36·7	37·1	34·9
25 to <30 kg/m^2^	48·3	50·0	47·8	47·8	47·6	49·1
30 to <35 kg/m^2^	12·6	12·8	14·3	12·3	12·1	12·8
≥35 kg/m^2^	3·3	3·9	3·0	3·2	3·2	3·2
Education (%)						
≤High school	27·4	30·7	27·5	27·0	26·1	27·9
Vocational school/some college	30·9	31·3	31·1	30·6	30·1	31·9
≥Graduated college	41·7	38·0	41·4	42·4	43·8	40·2
Marital status (%)						
Married	78·7	74·5	76·9	78·5	80·4	79·7
Separated/divorced	11·8	14·2	12·9	12·1	10·1	11·2
Widowed	1·9	2·6	1·8	1·9	2·0	1·6
Never married	7·7	8·7	8·4	7·5	7·5	7·6
Smoking status (%)						
Never	35·4	32·1	35·9	36·2	36·5	34·0
Past	49·2	52·5	47·8	49·3	48·0	49·4
Current	15·4	15·3	16·3	14·5	15·5	16·6
Physical activity† (h/d)						
Mean	1·5	1·4	1·6	1·5	1·5	1·4
sd	1·5	1·5	1·6	1·5	1·6	1·5
Multivitamin use (%)	48·1	50·9	48·1	48·3	48·0	47·0
Energy intake (kcal/d)‡						
Mean	2450·0	2342·6	2436·2	2482·4	2456·7	2434·7
sd	1038·4	1022·1	1074·6	1049·1	1034·6	1011·7
Alcohol intake (g/d)						
Mean	15·0	16·0	15·7	15·3	14·3	14·3
sd	28·7	32·9	27·1	27·9	27·7	29·6
Women (*n*)	30 456	2664	3187	11 671	5628	7306
Age (years)						
Mean	55·8	57·1	56·6	56·1	55·6	55·6
sd	7·1	7·2	7·2	7·1	7·1	6·9
Race/ethnicity (%)						
African American	12·0	15·4	14·0	12·7	10·4	9·9
Native Hawaiian	8·3	7·6	8·4	8·5	8·7	7·8
Japanese American	32·0	23·0	25·8	30·6	34·7	37·9
Latino	18·9	20·6	19·3	17·3	18·2	21·2
White	28·9	33·4	32·5	30·8	28·0	23·2
Body weight (kg)						
Mean	66·7	68·5	67·8	66·7	66·1	66·1
sd	14·6	14·9	15·0	14·4	14·4	14·6
BMI (%)						
18·5 to <25 kg/m^2^	51·0	46·1	49·4	51·3	52·5	51·9
25 to <30 kg/m^2^	30·9	32·9	30·8	31·5	30·2	29·9
30 to <35 kg/m^2^	12·2	13·7	13·0	11·7	11·9	12·3
≥35 kg/m^2^	5·9	7·3	6·8	5·6	5·5	5·9
Education (%)						
≤High school	33·8	37·1	35·1	32·9	32·3	34·4
Vocational school/some college	32·2	31·9	32·8	32·6	31·9	31·4
≥Graduated college	34·1	31·0	32·1	34·5	35·8	34·1
Marital status (%)						
Married	67·0	63·9	64·3	66·9	68·5	68·3
Separated/divorced	18·5	20·2	20·3	18·1	18·0	18·1
Widowed	8·5	10·0	9·0	8·8	8·0	7·5
Never married	6·0	5·9	6·4	6·2	5·5	6·1
Smoking status (%)						
Never	58·2	53·8	56·3	57·3	59·6	61·0
Past	29·6	32·2	31·4	30·2	29·4	27·1
Current	12·2	14·0	12·3	12·5	11·0	11·9
Physical activity† (h/d)						
Mean	1·2	1·2	1·3	1·2	1·2	1·1
sd	1·3	1·3	1·3	1·3	1·3	1·2
Multivitamin use (%)	54·0	55·9	56·6	53·8	54·0	52·3
MHT use (%)	49·2	53·3	50·5	49·8	48·6	46·7
Energy intake (kcal/d)‡						
Mean	1960·5	1887·3	1956·7	1979·8	1989·8	1935·3
sd	877·5	866·2	875·8	875·5	876·1	884·5
Alcohol intake (g/d)						
Mean	4·6	5·2	5·3	5·1	4·3	3·4
sd	13·4	13·8	14·3	14·2	13·3	11·5

MHT, menopausal hormone therapy.

* Greatest decline: ≥1 sd decrease; moderate decline: 0·5 to < 1 sd decrease; stable: < 0·5 sd change; moderate increase: 0·5 to < 1 sd increase; greatest increase: ≥ 1 sd increase.

† Hours in moderate vigorous activity per d.

‡ To convert kcal to kJ, multiply by 4·84.

Table 2 shows changes in body weight according to the categories of diet quality changes over 10 years after adjusting for the covariates. Compared with those who maintained stable dietary score, individuals with the greatest increase had significantly less weight gain: 0·55 (aMED) to 1·17 kg (DASH) less for men and 0·62 (aMED) to 1·31 kg (DASH) less for women. On the contrary, individuals with the greatest decrease in DQI had significantly greater weight gain: 0·41 (aMED) to 1·11 kg (DASH) more for men and 0·59 (AHEI-2010) to 1·08 kg (DASH) more for women, except for the HEI-2015 in which no significant weight change was found in men with the greatest decrease. When examining continuous DQI changes, each 1 sd increment was significantly associated with 0·40 (aMED) to 0·68 kg (DASH) less weight gain for men and 0·45 (aMED) to 0·74 kg (DASH) less weight gain for women (Table 2).

**Table 2.** Change in body weight according to change in diet quality over 10 years in the Multiethnic Cohort Study (Mean values and 95 % confidence intervals)

Diet quality change*	Men (*n* 23 521)			Women (*n* 30 456)		
	*n*	Weight change (kg)†		*n*	Weight change (kg)†	
		Mean	95 % CI		Mean	95 % CI
HEI-2015						
Greatest decline	1838	−0·02	−0·36, 0·33	2664	0·79	0·49, 1·10
Moderate decline	2452	0·14	−0·16, 0·44	3187	0·39	0·11, 0·67
Stable	9033	Ref.		11 671	Ref.	
Moderate increase	4320	−0·39	−0·63, −0·14	5628	−0·17	−0·40, 0·06
Greatest increase	5878	−0·98	−1·21, −0·75	7306	−0·85	−1·07, −0·63
Per 1 sd increase‡	23 521	−0·48	−0·58, −0·38	30 456	−0·54	−0·63, −0·45
AHEI-2010						
Greatest decline	2229	0·51	0·18, 0·83	3053	0·59	0·30, 0·89
Moderate decline	2717	0·26	−0·03, 0·55	3549	0·30	0·04, 0·57
Stable	9108	Ref.		11 717	Ref.	
Moderate increase	4101	−0·36	−0·60, −0·11	5364	−0·61	−0·84, −0·38
Greatest increase	5366	−0·94	−1·18, −0·70	6773	−0·99	−1·21, −0·76
Per 1 sd increase‡	23 521	−0·51	−0·61, −0·41	30 456	−0·58	−0·67, −0·49
aMED						
Greatest decline	4280	0·41	0·13, 0·70	5558	0·60	0·34, 0·87
Moderate decline	4121	0·44	0·16, 0·71	5337	0·26	0·01, 0·52
Stable	5327	Ref.		6856	Ref.	
Moderate increase	4568	−0·23	−0·49, 0·04	5829	−0·16	−0·41, 0·09
Greatest increase	5225	−0·55	−0·83, −0·28	6876	−0·62	−0·87, −0·37
Per 1 sd increase‡	23 521	−0·40	−0·51, −0·29	30 456	−0·45	−0·55, −0·34
DASH						
Greatest decline	1626	1·11	0·75, 1·47	2293	1·08	0·75, 1·41
Moderate decline	2240	0·58	0·27, 0·89	2931	0·28	−0·01, 0·57
Stable	10 968	Ref.		14 069	Ref.	
Moderate increase	3895	−0·43	−0·68, −0·19	5098	−0·51	−0·74, −0·28
Greatest increase	4792	−1·17	−1·40, −0·93	6065	−1·31	−1·54, −1·09
Per 1 sd increase‡	23 521	−0·68	−0·78, −0·58	30 456	−0·74	−0·83, −0·64

HEI-2015, Healthy Eating Index-2015; Ref., reference; AHEI-2010, Alternative Healthy Eating Index-2010; aMED, alternate Mediterranean Diet score; DASH, Dietary Approaches to Stop Hypertension.

* Greatest decline: ≥ 1 sd decrease; moderate decline: 0·5 to < 1 sd decrease; stable: < 0·5 sd change; moderate increase: 0·5 to < 1 sd increase; greatest increase: ≥ 1 sd increase.

† Values are means and 95 % CI adjusted for the following covariates: race/ethnicity; at baseline, age, BMI, education, marital status, smoking status, multivitamin use, diet quality score and, for women only, menopausal hormone therapy use; at baseline and change between the two surveys, physical activity and energy intake. For HEI-2015 and DASH, further adjusted for alcohol intake at baseline and change between the two surveys.

‡ All *P* values for weight change per 1 sd increase as *P*_for trend_ are less than 0·001.

In the analysis stratified by race/ethnicity, baseline age and baseline BMI, we found significantly less weight gain per 1 sd increase in the DQI for most subgroups (Table 3). In the racial/ethnic-specific analysis, however, African Americans showed no significant weight change per 1 sd increase in HEI-2015 (*P*_for heterogeneity_ by race/ethnicity: 0·046 for men and 0·001 for women). African Americans also showed no significant weight change with increase in AHEI-2010 (men only), aMED (men and women) and DASH (women only). In Native Hawaiian men, less weight change was found with increase in DASH but not for HEI-2015, AHEI-2010 and aMED. The inverse association between increase in diet quality and weight change was stronger in the younger age groups (45–59 years, 0·59–0·96 kg less for men and 0·85–1·22 kg less for women) than in the older group (60–69 years, 0·22–0·34 kg less for men and 0·15–0·22 kg less for women) in both men and women. The inverse association was also stronger in individuals with higher BMI (≥ 35 kg/m^2^, 1·46–2·04 kg less for men and 0·89–1·75 kg less for women) at baseline than in those with normal BMI (18·5– < 25 kg/m^2^, 0·25–0·40 kg less for men and 0·26–0·44 kg less for women).

**Table 3.** Change in body weight per 1 sd increase of diet quality over 10 years by race/ethnicity and baseline age and BMI in the Multiethnic Cohort Study (Mean values and 95 % confidence intervals)

Subgroups	*n*	Weight change (kg)*							
		HEI-2015		AHEI-2010		aMED		DASH	
		Mean	95 % CI	Mean	95 % CI	Mean	95 % CI	Mean	95 % CI
Men									
Race/ethnicity									
African American	1657	−0·04	−0·49, 0·41	−0·25	−0·72, 0·23	−0·03	−0·54, 0·48	−0·68	−1·16, −0·21
Native Hawaiian	1861	−0·28	−0·71, 0·16	−0·44	−0·90, 0·03	−0·25	−0·77, 0·26	−0·64	−1·08, −0·19
Japanese American	8265	−0·50	−0·63, −0·38	−0·49	−0·62, −0·36	−0·39	−0·53, −0·24	−0·56	−0·69, −0·44
Latino	4867	−0·46	−0·68, −0·24	−0·40	−0·62, −0·17	−0·41	−0·66, −0·16	−0·73	−0·96, −0·50
White	6871	−0·64	−0·83, −0·44	−0·73	−0·92, −0·54	−0·54	−0·75, −0·33	−0·85	−1·05, −0·64
*P*_for heterogeneity_		0·046		0·048		0·72		0·73	
Baseline age									
45–49 years	6064	−0·74	−0·94, −0·54	−0·74	−0·95, −0·53	−0·59	−0·82, −0·36	−0·96	−1·16, −0·76
50–59 years	9822	−0·59	−0·75, −0·44	−0·57	−0·73, −0·42	−0·52	−0·69, −0·35	−0·78	−0·93, −0·62
60–69 years	7635	−0·10	−0·26, 0·06	−0·22	−0·39, −0·06	−0·10	−0·28, 0·08	−0·34	−0·50, −0·17
*P*_for heterogeneity_		<0·001		0·002		0·072		<0·001	
Baseline BMI									
18·5 to < 25 kg/m^2^	8435	−0·27	−0·39, −0·15	−0·25	−0·37, −0·12	−0·28	−0·42, −0·15	−0·40	−0·53, −0·28
25 to <30 kg/m^2^	11 350	−0·57	−0·70, −0·44	−0·65	−0·78, −0·51	−0·44	−0·58, −0·29	−0·77	−0·90, −0·63
30 to < 35 kg/m^2^	2970	−0·44	−0·82, −0·06	−0·40	−0·79, −0·01	−0·40	−0·84, 0·04	−0·77	−1·16, −0·37
≥35 kg/m^2^	766	−1·46	−2·51, −0·41	−1·91	−3·08, −0·74	−0·94	−2·19, 0·30	−2·04	−3·14, −0·95
*P*_for heterogeneity_		<0·001		<0·001		0·20		<0·001	
Women									
Race/ethnicity									
African American	3645	−0·09	−0·43, 0·25	−0·43	−0·77, −0·08	0·03	−0·35, 0·42	−0·26	−0·61, 0·10
Native Hawaiian	2526	−0·69	−1·07, −0·32	−0·65	−1·04, −0·25	−0·54	−0·99, −0·09	−0·97	−1·35, −0·59
Japanese American	9730	−0·42	−0·54, −0·31	−0·43	−0·56, −0·31	−0·35	−0·48, −0·21	−0·55	−0·66, −0·43
Latino	5760	−0·70	−0·91, −0·50	−0·71	−0·93, −0·49	−0·65	−0·90, −0·40	−0·96	−1·19, −0·74
White	8795	−0·74	−0·92, −0·56	−0·70	−0·87, −0·52	−0·62	−0·82, −0·43	−0·94	−1·13, −0·76
*P*_for heterogeneity_		0·001		0·78		0·31		0·038	
Baseline age									
45–49 years	7671	−0·87	−1·06, −0·68	−0·88	−1·08, −0·69	−0·85	−1·08, −0·63	−1·22	−1·41, −1·02
50–59 years	12 568	−0·61	−0·75, −0·47	−0·63	−0·78, −0·49	−0·55	−0·71, −0·38	−0·80	−0·95, −0·65
60–69 years	10 217	−0·15	−0·29, −0·00	−0·17	−0·32, −0·02	0·02	−0·14, 0·19	−0·22	−0·37, −0·07
*P*_for heterogeneity_		<0·001		<0·001		0·013		<0·001	
Baseline BMI									
18·5 to < 25 kg/m^2^	15 530	−0·28	−0·38, −0·19	−0·29	−0·39, −0·19	−0·26	−0·37, −0·15	−0·44	−0·55, −0·34
25 to < 30 kg/m^2^	9417	−0·58	−0·74, −0·42	−0·61	−0·78, −0·45	−0·56	−0·75, −0·38	−0·80	−0·97, −0·63
30 to < 35 kg/m^2^	3712	−0·79	−1·11, −0·46	−0·93	−1·28, −0·59	−0·56	−0·96, −0·16	−1·07	−1·42, −0·72
≥35 kg/m^2^	1797	−1·44	−2·07, −0·82	−1·75	−2·40, −1·10	−0·89	−1·66, −0·11	−1·74	−2·39, −1·09
*P*_for heterogeneity_		<0·001		<0·001		0·42		<0·001	

HEI-2015, Healthy Eating Index-2015; AHEI-2010, Alternative Healthy Eating Index-2010; aMED, alternate Mediterranean Diet score; DASH, Dietary Approaches to Stop Hypertension.

* Values are means and 95 % CI adjusted for the following covariates: race/ethnicity; at baseline, age, BMI, education, marital status, smoking status, multivitamin use, diet quality score and, for women only, menopausal hormone therapy use; at baseline and change between the two surveys, physical activity and energy intake. For HEI-2015 and DASH, further adjusted for alcohol intake at baseline and change between the two surveys.

**Fig. 1.**
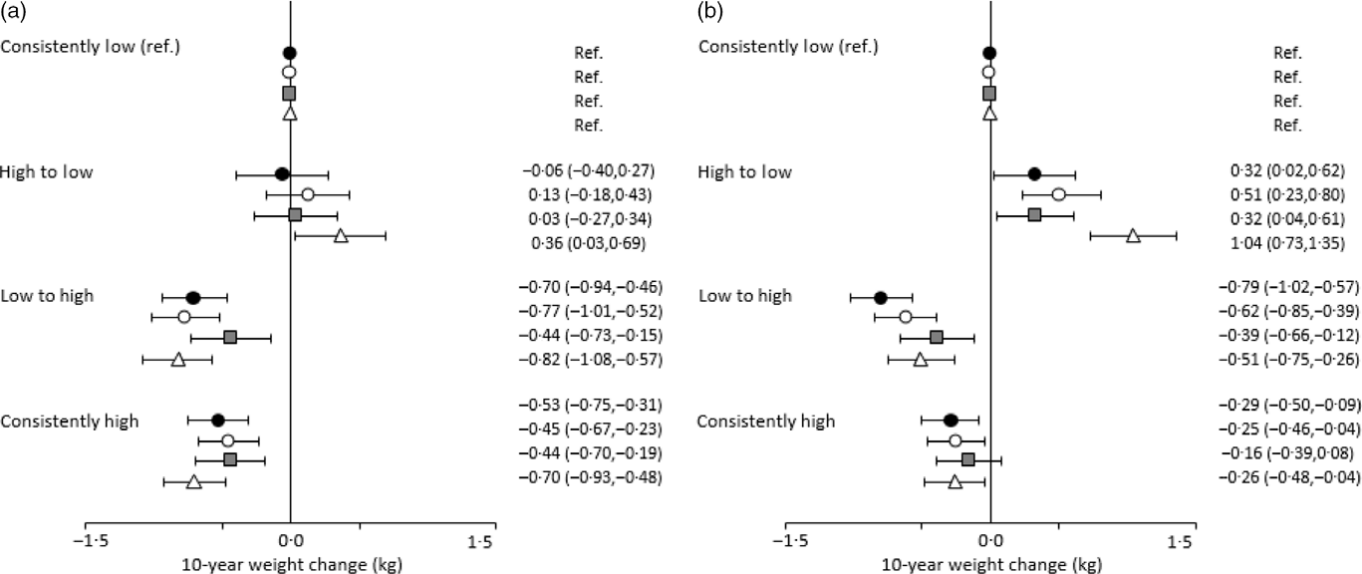
Change in diet quality and change in body weight over 10 years in the Multiethnic Cohort Study: (a) men and (b) women. Values are mean weight changes with 95 % confidence intervals adjusted for the following covariates: race/ethnicity; at baseline, age, BMI, education, marital status, smoking status, multivitamin use and, for women only, menopausal hormone therapy use; at baseline and change between the two surveys, physical activity and energy intake. For Healthy Eating Index-2015 (HEI-2015) and Dietary Approaches to Stop Hypertension (DASH), further adjusted for alcohol intake at baseline and change between the two surveys. Changes in diet quality were categorised into four groups based on the median scores of each index at baseline as follows: (1) consistently low (reference (ref.) group), below the median value at both surveys, (2) high to low, from above to below the median, (3) low to high, from below to above the median, and (4) consistently high, above the median value at both surveys. 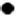, HEI-2015; 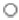, Alternative Healthy Eating Index-2010; 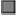, alternate Mediterranean Diet score; 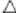, DASH.

Fig. 1 shows the relation between diet quality trajectories over 10 years and concurrent change in body weight. Compared with those with consistently low diet quality, men with consistently high diet quality had a 0·44–0·70 kg smaller weight gain. Improving diet quality was also associated with a smaller weight gain (0·44–0·82 kg) in men. On the other hand, a decrease in DASH score was associated with a significantly higher weight gain of 0·36 (95 % CI 0·03, 0·69) kg in men. A similar tendency was observed for women. Women with consistently high diet quality over 10 years had a 0·25–0·29 kg smaller weight gain, compared with those with consistently low diet quality. An inverse association was also found in women with improvement in diet quality (0·39–0·79 kg smaller weight gain). Women with decreased diet quality had a significantly higher weight gain (0·32–1·04 kg) compared with those with consistently low diet quality.

## Discussion

The present study examined the associations between changes in diet quality assessed by four predefined DQI and changes in body weight over 10 years in a large multiethnic population. Maintaining a high-quality diet and improvement in diet quality were associated with less weight gain both in men and women. Smaller weight gain with improvement in diet quality was found in most subgroups by race/ethnicity, baseline age and baseline BMI. The inverse association was stronger in younger age and higher BMI groups, compared with older and lower BMI groups, respectively.

Several recent studies have shown an inverse association between diet quality at baseline and subsequent weight change^(6–9)^. The European Prospective Investigation into Cancer and Nutrition-Physical Activity, Nutrition, Alcohol Consumption, Cessation of Smoking, Eating Out of Home, and Obesity project reported that individuals with high adherence to the Mediterranean dietary pattern showed 0·16 kg less weight gain during the 5-year follow-up, compared with those with low adherence^(6)^. Data from the Seguimiento Universidad de Navarra (University of Navarra Follow-Up) study also showed that high adherence to the Mediterranean dietary pattern was associated with 0·059 kg/year smaller weight gain during the mean follow-up of 5·7 years^(7)^. In a cohort in France, higher scores in several dietary indexes (the French Programme National Nutrition Santé-Guidelines Score, the Dietary Guidelines for Americans Index, the Diet quality Index-International, the Mediterranean Diet Scale and the relative Mediterranean Diet Score) were significantly associated with less weight gain by 0·84–2·17 % of the baseline weight for men but not for women during 13-year follow-up^(8)^. Additionally, a prospective study in Australia showed that men who had high adherence to the Australian dietary guideline gained less BMI compared with men who had low adherence (0·05 *v*. 0·11 kg/m^2^ per year, *P* = 0·01) during 15-year follow-up^(9)^.

However, few studies have reported on the association between longitudinal diet quality change and concurrent body weight or composition change. In the Nurses’ Health Study and Health Professionals Follow-up Study^(10)^, a 1 sd increase in three DQI in 4-year periods over 20 years was associated with less weight gain by 0·47 kg for AHEI-2010, 0·42 kg for DASH and 0·23 kg for aMED in men and women combined. The association was stronger in the younger cohort (Nurses’ Health Study II) than the older cohort (Nurses’ Health Study I and Health Professionals Follow-up Study), and among overweight (BMI ≥ 25 kg/m^2^) than normal-weight (BMI < 25 kg/m^2^) individuals^(10)^, which was consistent with our results. In the Women’s Health Initiative Observational Study, a 10 % increase in the four DQI was associated with smaller increase in waist circumference over 3 years by 0·43 cm for AHEI-2010, 0·40 cm for HEI-2010, 0·28 cm for DASH and 0·07 cm for a MED^(21)^. In the present study, relatively weaker associations were observed with aMED, compared with the other DQI, which was also found in the Nurses’ Health Study and Health Professionals Follow-up Study^(10)^ and the Women’s Health Initiative Observational Study^(21)^. These may be partly because the different scoring system across the DQI. For instance, HEI-2015 (0–100 points), AHEI-2010 (0–110) and DASH (8–40) have wider ranges of total scores compared with aMED (0–9). Nevertheless, as all of these DQI emphasise healthful diet, the direction of association between improving diet quality and less weight gain was consistent across all the analysed indexes.

In the current study, smaller weight gain with improvement in diet quality was found across racial/ethnic groups in general, but it was less apparent in African Americans especially for HEI-2015. The explanation for this inconsistency is not clear. However, African American men and women had the highest HEI-2015 scores at baseline and showed the smallest increase over 10 years, while their mean scores for the other indexes at baseline were similar to or less than the overall means (online Supplementary Table S1). Unlike HEI-2015, improvement in DASH (men) and AHEI-2010 (women) was significantly associated with less weight gain in African Americans. The racial/ethnic differences in the associations might be influenced by differences in components and scoring systems in each of the DQI, but this warrants further investigation.

The association between improving diet quality and less weight gain was stronger in the younger age groups (45–59 *v*. 60–69 years at baseline) in the present study. This may be partially due to weight change patterns over 10 years varied by age group. Among individuals aged 45–49 years at cohort entry, mean body weight increased by 2·8 (sd 7·1) kg for men and 3·6 (sd 7·7) kg for women, whereas it decreased by 0·4 (sd 6·1) kg for men and 0·3 (sd 6·4) kg for women aged 60–69 years at cohort entry. In addition, the oldest age group had higher DQI scores at baseline^(22)^ and showed smaller increases in the DQI over 10 years in general^(23)^, compared with the younger age groups.

In previous studies in the MEC, higher BMI at cohort entry was associated with higher risk of mortality^(24)^, pancreatic cancer for men^(25)^, breast cancer incidence for women^(26)^ and breast cancer-specific mortality among women diagnosed with incident breast cancer^(27)^. In studies examining weight both at age 21 years and cohort entry, adult obesity and increase in adiposity during adulthood were related to higher risk for prostate carcinogenesis in men^(28)^, breast cancer in women^(26)^, endometrial cancer in women^(29)^ and diabetes both in men and women^(30)^. Thus, maintaining a healthy weight throughout adulthood is important for preventing premature death and chronic disease. In the MEC, the highest BMI group (≥35 kg/m^2^) showed lower DQI scores at baseline and smaller increases in the DQI scores over 10 years, compared with the lower BMI groups^(22,23)^. Nonetheless, in the current study, improving diet quality seemed to benefit the highest BMI group more than the lower BMI groups. If confirmed, this would strengthen the incentive for individuals who need weight control to adopt a healthy dietary pattern.

The present study has several strengths including a prospective design, a large number of participants in a population-based multiethnic cohort, repeated dietary assessment over 10 years by a validated QFFQ and comprehensive information on a wide range of potential covariates. However, there are several limitations that need to be considered. First, body weight assessments for both surveys were based on self-report and thus misclassification of weight change may have occurred, although a high correlation between self-reported and measured weight was observed in both White (*r* 0·98) and Japanese Americans (*r* 0·99) in a study of sixty female MEC participants^(31)^ as well as in other study populations^(32–34)^. In addition, systematic errors according to sex, ethnicity and obesity status have been reported^(35)^, despite strong correlations between self-reported and measured weight. Second, dietary data based on a self-administered QFFQ are subject to non-differential measurement error, which commonly occurs in a cohort study resulting in attenuated risk estimates^(36)^. Third, although participants who had previous cancer or heart disease at either survey were excluded from the current analysis, we could not completely rule out any changes in diet quality or body weight due to underlying illness. Finally, the current findings are based on the MEC participants who completed the 10-year follow-up survey (45 % of total) and were somewhat different from non-respondents as described in the ‘Methods’ section, with further restriction to those (approximately 54 000) with normal or higher BMI and without cancer or heart disease. Thus, a selection bias that would limit generalisability cannot be excluded.

### Conclusions

In a multiethnic population, 10-year improvement in diet quality was associated with a smaller weight gain overall, which varied by race/ethnicity, age and BMI at baseline. Our findings suggest maintaining a high-quality diet and improving diet quality over time may be helpful in preventing excessive weight gain.

## Supplementary material

For supplementary material accompanying this paper visit http://dx.doi.org/10.1017/S000711452100012X. click here to view supplementary material
