# Supplementary material for: Changes in diet quality and body weight over 10 years: the Multiethnic Cohort Study
Source: Br J Nutr. 2021 Jan 14;126(9):1389–97. doi: 10.1017/S000711452100012X (PMC8505709; doi:10.1017/S000711452100012X)
Supplement: Supplementary file 1 [file S000711452100012Xsup001.docx]

Supplemental Table 1. Body weight and diet quality index scores at baseline and 10-year follow-up surveys in men and women overall and by race/ethnicity in the Multiethnic Cohort Study

|  | Men (n = 23,521) | | | Women (n = 30,456) | | |
| --- | --- | --- | --- | --- | --- | --- |
|  | Baseline | 10-year  follow-up | Change | Baseline | 10-year  follow-up | Change |
| Body weight (kg) |  |  |  |  |  |  |
| All | 80.3 ± 14.1 | 81.5 ± 15.4 | 1.2 ± 6.8 | 66.7 ± 14.6 | 68.2 ± 15.6 | 1.5 ± 7.2 |
| African American | 87.2 ± 14.5 | 88.4 ± 16.0 | 1.3 ± 8.0 | 77.3 ± 16.0 | 79.0 ± 17.1 | 1.7 ± 9.3 |
| Native Hawaiian | 90.1 ± 16.9 | 91.2 ± 18.0 | 1.1 ± 8.9 | 74.1 ± 16.4 | 76.1 ± 17.1 | 2.0 ± 8.7 |
| Japanese American | 73.1 ± 10.9 | 73.6 ± 11.7 | 0.5 ± 5.2 | 57.9 ± 9.5 | 58.7 ± 10.4 | 0.8 ± 5.0 |
| Latino | 81.5 ± 12.6 | 82.7 ± 13.9 | 1.2 ± 6.8 | 69.4 ± 12.9 | 71.0 ± 13.8 | 1.6 ± 7.2 |
| White | 83.9 ± 13.5 | 85.9 ± 15.1 | 2.0 ± 7.2 | 68.2 ± 13.7 | 70.3 ± 14.9 | 2.1 ± 7.6 |
| HEI-2015 |  |  |  |  |  |  |
| All | 65.2 ± 10.3 | 68.8 ± 10.6 | 3.5 ± 9.8 | 69.0 ± 10.3 | 72.3 ± 10.6 | 3.3 ± 9.9 |
| African American | 68.3 ± 10.1 | 69.9 ± 10.3 | 1.6 ± 10.1 | 71.5 ± 10.2 | 73.3 ± 10.5 | 1.8 ± 10.0 |
| Native Hawaiian | 63.4 ± 10.4 | 67.5 ± 10.9 | 4.0 ± 9.9 | 67.2 ± 10.5 | 70.5 ± 10.9 | 3.3 ± 9.8 |
| Japanese American | 63.3 ± 10.2 | 68.6 ± 10.8 | 5.3 ± 9.6 | 68.0 ± 10.2 | 72.7 ± 10.6 | 4.7 ± 9.6 |
| Latino | 64.4 ± 9.4 | 67.3 ± 10.0 | 2.9 ± 9.7 | 67.1 ± 9.8 | 70.6 ± 10.5 | 3.6 ± 10.4 |
| White | 68.0 ± 10.2 | 70.2 ± 10.5 | 2.2 ± 9.5 | 71.0 ± 10.0 | 73.0 ± 10.4 | 2.0 ± 9.6 |
| AHEI-2010 |  |  |  |  |  |  |
| All | 64.3 ± 9.9 | 67.1 ± 10.4 | 2.8 ± 10.0 | 65.4 ± 9.3 | 67.9 ± 10.0 | 2.5 ± 9.5 |
| African American | 63.7 ± 9.6 | 64.3 ± 9.7 | 0.6 ± 9.8 | 65.2 ± 9.3 | 65.7 ± 9.9 | 0.5 ± 9.7 |
| Native Hawaiian | 63.6 ± 9.6 | 67.7 ± 9.8 | 4.0 ± 9.7 | 64.9 ± 9.4 | 68.2 ± 9.4 | 3.4 ± 9.6 |
| Japanese American | 64.6 ± 10.0 | 69.4 ± 10.0 | 4.7 ± 9.8 | 66.3 ± 9.3 | 70.4 ± 9.0 | 4.0 ± 8.9 |
| Latino | 62.5 ± 9.1 | 62.8 ± 9.9 | 0.3 ± 10.0 | 63.2 ± 8.4 | 63.3 ± 9.5 | 0.1 ± 9.5 |
| White | 65.5 ± 10.3 | 67.9 ± 10.6 | 2.4 ± 9.9 | 66.2 ± 9.5 | 68.9 ± 10.2 | 2.8 ± 9.6 |
| aMED |  |  |  |  |  |  |
| All | 4.2 ± 1.8 | 4.4 ± 1.8 | 0.1 ± 1.9 | 4.1 ± 1.8 | 4.3 ± 1.8 | 0.1 ± 1.9 |
| African American | 4.2 ± 1.8 | 4.1 ± 1.8 | -0.1 ± 1.9 | 4.1 ± 1.8 | 4.1 ± 1.9 | -0.0 ± 1.9 |
| Native Hawaiian | 4.3 ± 1.8 | 4.4 ± 1.8 | 0.1 ± 1.8 | 4.3 ± 1.8 | 4.3 ± 1.8 | 0.0 ± 1.9 |
| Japanese American | 4.2 ± 1.8 | 4.4 ± 1.8 | 0.2 ± 1.8 | 4.2 ± 1.8 | 4.4 ± 1.7 | 0.2 ± 1.8 |
| Latino | 4.0 ± 1.7 | 4.2 ± 1.8 | 0.1 ± 1.9 | 3.9 ± 1.7 | 4.1 ± 1.8 | 0.2 ± 1.9 |
| White | 4.4 ± 1.8 | 4.5 ± 1.9 | 0.0 ± 1.9 | 4.2 ± 1.8 | 4.3 ± 1.8 | 0.1 ± 1.9 |
| DASH |  |  |  |  |  |  |
| All | 23.8 ± 4.5 | 25.1 ± 4.5 | 1.3 ± 4.0 | 24.1 ± 4.4 | 25.3 ± 4.4 | 1.2 ± 4.0 |
| African American | 23.8 ± 4.2 | 24.3 ± 4.2 | 0.6 ± 4.1 | 23.8 ± 4.3 | 24.3 ± 4.4 | 0.5 ± 4.1 |
| Native Hawaiian | 22.6 ± 4.4 | 24.0 ± 4.5 | 1.4 ± 4.1 | 22.7 ± 4.5 | 24.1 ± 4.6 | 1.3 ± 4.3 |
| Japanese American | 22.4 ± 4.5 | 24.4 ± 4.6 | 2.0 ± 4.1 | 23.1 ± 4.4 | 24.8 ± 4.4 | 1.7 ± 4.0 |
| Latino | 24.4 ± 4.0 | 25.3 ± 4.1 | 0.9 ± 4.0 | 24.4 ± 4.1 | 25.4 ± 4.3 | 1.0 ± 4.1 |
| White | 25.5 ± 4.3 | 26.3 ± 4.3 | 0.8 ± 3.8 | 25.4 ± 4.2 | 26.4 ± 4.2 | 1.0 ± 3.9 |

HEI-2015, Healthy Eating Index-2015; AHEI-2010, Alternative Healthy Eating Index-2010; aMED, alternate Mediterranean Diet score; DASH, Dietary Approaches to Stop Hypertension

Values are mean ± SD.

Supplemental Table 2. Baseline characteristics by change in Alternative Healthy Eating Index-2010 over 10 years in the Multiethnic Cohort Study

| Characteristics | Change in Alternative Healthy Eating Index-2010^a^ | | | | |
| --- | --- | --- | --- | --- | --- |
|  | Greatest decline | Moderate decline | Stable | Moderate increase | Greatest increase |
| Men, n | 2,229 | 2,717 | 9,108 | 4,101 | 5,366 |
| Age, y | 56.6 ± 6.9 | 56.3 ± 7.1 | 55.8 ± 7.1 | 55.1 ± 7.1 | 54.7 ± 6.9 |
| Race/ethnicity (%) |  |  |  |  |  |
| African American | 9.5 | 9.3 | 7.1 | 6.5 | 5.2 |
| Native Hawaiian | 6.0 | 6.3 | 8.0 | 9.0 | 8.5 |
| Japanese American | 22.7 | 26.8 | 34.0 | 37.9 | 44.3 |
| Latino | 31.2 | 26.2 | 21.5 | 17.5 | 14.7 |
| White | 30.6 | 31.4 | 29.3 | 29.1 | 27.4 |
| Body weight, kg | 81.4 ± 14.4 | 80.9 ± 13.5 | 80.2 ± 14.2 | 80.3 ± 14.2 | 79.7 ± 14.0 |
| Body mass index (%) |  |  |  |  |  |
| 18.5-<25 kg/m^2^ | 33.5 | 34.7 | 36.4 | 36.6 | 36.0 |
| 25-<30 kg/m^2^ | 49.8 | 48.7 | 48.1 | 47.2 | 48.5 |
| 30-<35 kg/m^2^ | 13.2 | 14.1 | 12.1 | 13.1 | 12.2 |
| ≥35 kg/m^2^ | 3.5 | 2.5 | 3.4 | 3.2 | 3.3 |
| Education (%) |  |  |  |  |  |
| ≤High school | 34.0 | 29.1 | 27.0 | 25.7 | 25.6 |
| Vocational school/some college | 29.8 | 30.4 | 30.2 | 31.7 | 32.3 |
| ≥Graduated college | 36.2 | 40.6 | 42.7 | 42.6 | 42.0 |
| Marital status (%) |  |  |  |  |  |
| Married | 77.0 | 77.1 | 79.3 | 78.1 | 79.6 |
| Separated/divorced | 13.7 | 12.7 | 11.2 | 11.8 | 11.4 |
| Widowed | 1.9 | 2.1 | 2.1 | 1.8 | 1.5 |
| Never married | 7.5 | 8.1 | 7.5 | 8.3 | 7.5 |
| Smoking status (%) |  |  |  |  |  |
| Never | 33.9 | 34.2 | 37.1 | 36.4 | 32.8 |
| Past | 50.5 | 49.7 | 48.4 | 48.1 | 50.5 |
| Current | 15.6 | 16.1 | 14.5 | 15.5 | 16.7 |
| Physical activity^b^, h/d | 1.5 ± 1.6 | 1.5 ± 1.6 | 1.5 ± 1.5 | 1.5 ± 1.5 | 1.5 ± 1.5 |
| Multivitamin use (%) | 49.5 | 50.1 | 48.4 | 47.5 | 46.4 |
| Energy intake, kcal/d | 2527.0 ± 1117.6 | 2484.7 ± 1098.8 | 2457.1 ± 1035.8 | 2439.5 ± 996.9 | 2396.5 ± 1005.1 |
| Alcohol intake, g/d | 12.5 ± 19.2 | 12.7 ± 23.3 | 12.8 ± 25.4 | 15.5 ± 29.2 | 20.3 ± 37.2 |
|  |  |  |  |  |  |
| Women, n | 3,053 | 3,549 | 11,717 | 5,364 | 6,773 |
| Age, y | 57.0 ± 7.0 | 56.7 ± 7.1 | 56.2 ± 7.1 | 55.4 ± 7.2 | 54.3 ± 6.9 |
| Race/ethnicity (%) |  |  |  |  |  |
| African American | 17.0 | 15.2 | 12.0 | 10.2 | 9.3 |
| Native Hawaiian | 7.0 | 7.4 | 8.2 | 8.5 | 9.4 |
| Japanese American | 19.5 | 25.8 | 31.9 | 36.6 | 37.2 |
| Latino | 28.5 | 23.6 | 19.6 | 15.9 | 13.2 |
| White | 28.0 | 28.0 | 28.3 | 28.8 | 30.9 |
| Body weight, kg | 69.1 ± 15.1 | 67.4 ± 14.5 | 66.6 ± 14.6 | 66.0 ± 14.4 | 65.9 ± 14.3 |
| Body mass index (%) |  |  |  |  |  |
| 18.5-<25 kg/m^2^ | 44.2 | 49.2 | 50.7 | 53.1 | 53.8 |
| 25-<30 kg/m^2^ | 33.2 | 31.7 | 31.4 | 29.6 | 29.6 |
| 30-<35 kg/m^2^ | 15.4 | 12.9 | 11.9 | 11.5 | 11.4 |
| ≥35 kg/m^2^ | 7.3 | 6.2 | 5.9 | 5.8 | 5.2 |
| Education (%) |  |  |  |  |  |
| ≤High school | 41.5 | 38.4 | 34.6 | 31.8 | 28.1 |
| Vocational school/some college | 31.5 | 31.4 | 31.9 | 31.9 | 33.4 |
| ≥Graduated college | 26.9 | 30.2 | 33.5 | 36.3 | 38.6 |
| Marital status (%) |  |  |  |  |  |
| Married | 62.6 | 64.9 | 67.0 | 68.5 | 68.8 |
| Separated/divorced | 20.7 | 19.6 | 17.9 | 18.3 | 18.2 |
| Widowed | 10.3 | 9.6 | 8.8 | 7.3 | 7.2 |
| Never married | 6.4 | 5.9 | 6.2 | 5.9 | 5.7 |
| Smoking status (%) |  |  |  |  |  |
| Never | 53.6 | 56.8 | 59.8 | 59.3 | 57.3 |
| Past | 31.6 | 31.9 | 28.4 | 29.4 | 29.9 |
| Current | 14.9 | 11.4 | 11.8 | 11.4 | 12.8 |
| Physical activity^b^, h/d | 1.2 ± 1.3 | 1.2 ± 1.3 | 1.2 ± 1.3 | 1.2 ± 1.3 | 1.2 ± 1.3 |
| Multivitamin use (%) | 55.3 | 54.5 | 53.9 | 52.6 | 54.3 |
| MHT use (%) | 51.3 | 51.6 | 50.1 | 49.3 | 45.6 |
| Energy intake, kcal/d | 2078.7 ± 947.9 | 2006.5 ± 925.1 | 1983.5 ± 894.8 | 1938.8 ± 852.7 | 1860.3 ± 793.3 |
| Alcohol intake, g/d | 5.2 ± 11.9 | 4.3 ± 11.0 | 4.0 ± 11.8 | 4.6 ± 14.9 | 5.4 ± 16.2 |

MHT, menopausal hormone therapy

^a^ Greatest decline: ≥1 SD decrease; moderate decline: 0.5–<1 SD decrease; stable: <0.5 SD change; moderate increase: 0.5–<1 SD increase; greatest increase: ≥1 SD increase ^b^ Hours in moderate vigorous activity per day

Supplemental Table 3. Baseline characteristics by change in alternate Mediterranean Diet over 10 years in the Multiethnic Cohort Study

| Characteristics | Change in alternate Mediterranean Diet score^a^ | | | | |
| --- | --- | --- | --- | --- | --- |
|  | Greatest decline | Moderate decline | Stable | Moderate increase | Greatest increase |
| Men, n | 4,280 | 4,121 | 5,327 | 4,568 | 5,225 |
| Age, y | 56.1 ± 7.1 | 55.8 ± 7.2 | 55.6 ± 7.0 | 55.4 ± 7.0 | 55.2 ± 6.9 |
| Race/ethnicity (%) |  |  |  |  |  |
| African American | 8.6 | 7.5 | 6.7 | 6.3 | 6.4 |
| Native Hawaiian | 8.1 | 8.2 | 8.3 | 7.7 | 7.3 |
| Japanese American | 30.8 | 34.8 | 34.8 | 36.8 | 37.9 |
| Latino | 21.6 | 18.9 | 21.2 | 20.3 | 21.2 |
| White | 30.9 | 30.6 | 29.0 | 28.8 | 27.3 |
| Body weight, kg | 81.1 ± 14.5 | 80.7 ± 14.3 | 80.3 ± 14.0 | 80.0 ± 13.9 | 79.7 ± 13.8 |
| Body mass index (%) |  |  |  |  |  |
| 18.5-<25 kg/m^2^ | 35.3 | 35.8 | 35.6 | 36.4 | 36.2 |
| 25-<30 kg/m^2^ | 47.5 | 48.1 | 48.5 | 48.1 | 48.9 |
| 30-<35 kg/m^2^ | 13.5 | 12.3 | 13.0 | 12.7 | 11.8 |
| ≥35 kg/m^2^ | 3.8 | 3.8 | 3.0 | 2.9 | 3.0 |
| Education (%) |  |  |  |  |  |
| ≤High school | 28.4 | 27.7 | 28.0 | 26.9 | 26.2 |
| Vocational school/some college | 30.3 | 31.1 | 30.7 | 31.3 | 31.2 |
| ≥Graduated college | 41.3 | 41.2 | 41.2 | 41.8 | 42.6 |
| Marital status (%) |  |  |  |  |  |
| Married | 77.6 | 79.0 | 79.0 | 78.8 | 78.9 |
| Separated/divorced | 13.2 | 11.4 | 12.0 | 10.6 | 11.7 |
| Widowed | 2.0 | 1.7 | 1.9 | 1.8 | 1.9 |
| Never married | 7.2 | 8.0 | 7.1 | 8.8 | 7.5 |
| Smoking status (%) |  |  |  |  |  |
| Never | 35.3 | 36.7 | 35.6 | 35.8 | 33.8 |
| Past | 50.1 | 48.1 | 48.9 | 49.3 | 49.6 |
| Current | 14.6 | 15.2 | 15.5 | 14.9 | 16.7 |
| Physical activity^b^, h/d | 1.5 ± 1.5 | 1.5 ± 1.5 | 1.5 ± 1.5 | 1.5 ± 1.6 | 1.4 ± 1.5 |
| Multivitamin use (%) | 49.1 | 49.2 | 46.9 | 48.6 | 47.2 |
| Energy intake, kcal/d | 2697.4 ± 1079.5 | 2569.4 ± 1077.0 | 2472.7 ± 1070.6 | 2386.9 ± 1019.0 | 2185.2 ± 879.4 |
| Alcohol intake, g/d | 14.5 ± 27.1 | 15.0 ± 30.0 | 14.4 ± 27.0 | 15.7 ± 31.1 | 15.2 ± 28.2 |
|  |  |  |  |  |  |
| Women, n | 5,558 | 5,337 | 6,856 | 5,829 | 6,876 |
| Age, y | 56.6 ± 7.1 | 56.2 ± 7.1 | 56.0 ± 7.1 | 55.4 ± 7.1 | 54.9 ± 7.1 |
| Race/ethnicity (%) |  |  |  |  |  |
| African American | 14.2 | 12.3 | 11.1 | 11.4 | 11.3 |
| Native Hawaiian | 9.1 | 8.1 | 8.2 | 8.3 | 7.8 |
| Japanese American | 29.1 | 31.5 | 33.4 | 33.5 | 31.8 |
| Latino | 19.0 | 17.7 | 18.5 | 19.2 | 20.0 |
| White | 28.6 | 30.4 | 28.8 | 27.6 | 29.1 |
| Body weight, kg | 68.0 ± 15.0 | 67.0 ± 14.9 | 66.5 ± 14.3 | 66.4 ± 14.8 | 66.0 ± 13.9 |
| Body mass index (%) |  |  |  |  |  |
| 18.5-<25 kg/m^2^ | 47.5 | 50.3 | 51.7 | 51.8 | 53.0 |
| 25-<30 kg/m^2^ | 32.4 | 30.7 | 30.8 | 30.6 | 30.4 |
| 30-<35 kg/m^2^ | 13.2 | 12.8 | 11.8 | 11.4 | 12.0 |
| ≥35 kg/m^2^ | 6.9 | 6.2 | 5.7 | 6.3 | 4.7 |
| Education (%) |  |  |  |  |  |
| ≤High school | 36.4 | 33.8 | 33.6 | 33.3 | 32.3 |
| Vocational school/some college | 31.2 | 32.5 | 32.8 | 32.2 | 32.0 |
| ≥Graduated college | 32.4 | 33.7 | 33.7 | 34.6 | 35.7 |
| Marital status (%) |  |  |  |  |  |
| Married | 65.6 | 66.6 | 67.8 | 67.5 | 67.1 |
| Separated/divorced | 19.1 | 18.4 | 17.5 | 18.4 | 19.3 |
| Widowed | 9.6 | 8.4 | 9.0 | 7.9 | 7.5 |
| Never married | 5.7 | 6.6 | 5.8 | 6.2 | 6.1 |
| Smoking status (%) |  |  |  |  |  |
| Never | 56.3 | 57.7 | 59.0 | 58.9 | 58.8 |
| Past | 31.3 | 30.3 | 29.4 | 28.6 | 28.8 |
| Current | 12.5 | 12.1 | 11.6 | 12.5 | 12.4 |
| Physical activity^b^, h/d | 1.2 ± 1.3 | 1.2 ± 1.3 | 1.2 ± 1.3 | 1.2 ± 1.3 | 1.2 ± 1.3 |
| Multivitamin use (%) | 55.0 | 54.5 | 54.2 | 53.4 | 52.9 |
| MHT use (%) | 51.6 | 49.9 | 50.6 | 46.8 | 47.5 |
| Energy intake, kcal/d | 2214.6 ± 960.7 | 2041.9 ± 884.5 | 1983.0 ± 891.3 | 1895.5 ± 844.0 | 1724.4 ± 737.9 |
| Alcohol intake, g/d | 4.9 ± 14.0 | 4.5 ± 12.5 | 4.5 ± 13.4 | 4.4 ± 13.3 | 4.5 ± 13.8 |

MHT, menopausal hormone therapy

^a^ Greatest decline: ≥1 SD decrease; moderate decline: 0.5–<1 SD decrease; stable: <0.5 SD change; moderate increase: 0.5–<1 SD increase; greatest increase: ≥1 SD increase ^b^ Hours in moderate vigorous activity per day

Supplemental Table 4. Baseline characteristics by change in Dietary Approaches to Stop Hypertension over 10 years among 53,977 participants in the Multiethnic Cohort Study

| Characteristics | Change in Dietary Approaches to Stop Hypertension^a^ | | | | |
| --- | --- | --- | --- | --- | --- |
|  | Greatest decline | Moderate decline | Stable | Moderate increase | Greatest increase |
| Men, n | 1,626 | 2,240 | 10,968 | 3,895 | 4,792 |
| Age, y | 56.3 ± 7.3 | 56.1 ± 7.1 | 55.7 ± 7.1 | 55.3 ± 7.0 | 55.1 ± 6.9 |
| Race/ethnicity (%) |  |  |  |  |  |
| African American | 9.7 | 8.4 | 7.3 | 6.1 | 5.7 |
| Native Hawaiian | 7.5 | 7.6 | 7.8 | 7.7 | 8.6 |
| Japanese American | 25.6 | 28.1 | 32.5 | 39.8 | 43.7 |
| Latino | 25.3 | 21.7 | 21.0 | 20.5 | 18.1 |
| White | 31.9 | 34.2 | 31.3 | 26.0 | 23.8 |
| Body weight, kg | 81.4 ± 14.3 | 81.3 ± 14.5 | 80.4 ± 14.1 | 79.8 ± 13.8 | 79.8 ± 14.0 |
| Body mass index (%) |  |  |  |  |  |
| 18.5-<25 kg/m^2^ | 33.8 | 35.3 | 37.1 | 35.3 | 34.5 |
| 25-<30 kg/m^2^ | 50.1 | 48.5 | 47.2 | 49.1 | 49.2 |
| 30-<35 kg/m^2^ | 12.6 | 12.2 | 12.6 | 12.6 | 13.0 |
| ≥35 kg/m^2^ | 3.5 | 4.0 | 3.2 | 3.0 | 3.2 |
| Education (%) |  |  |  |  |  |
| ≤High school | 30.2 | 26.5 | 26.3 | 26.9 | 29.7 |
| Vocational school/some college | 30.8 | 30.7 | 30.3 | 31.9 | 31.7 |
| ≥Graduated college | 39.0 | 42.8 | 43.3 | 41.2 | 38.7 |
| Marital status (%) |  |  |  |  |  |
| Married | 76.6 | 77.8 | 78.7 | 78.7 | 79.7 |
| Separated/divorced | 12.4 | 12.6 | 12.4 | 10.9 | 10.5 |
| Widowed | 2.2 | 2.0 | 1.9 | 1.9 | 1.7 |
| Never married | 8.9 | 7.6 | 7.1 | 8.5 | 8.0 |
| Smoking status (%) |  |  |  |  |  |
| Never | 33.8 | 35.2 | 36.4 | 35.9 | 33.1 |
| Past | 51.7 | 50.1 | 48.5 | 48.1 | 50.3 |
| Current | 14.4 | 14.7 | 15.0 | 16.0 | 16.6 |
| Physical activity^b^, h/d | 1.6 ± 1.6 | 1.5 ± 1.5 | 1.5 ± 1.5 | 1.5 ± 1.6 | 1.4 ± 1.5 |
| Multivitamin use (%) | 52.3 | 49.8 | 48.3 | 47.8 | 45.5 |
| Energy intake, kcal/d | 2637.1 ± 1170.3 | 2569.3 ± 1097.7 | 2473.0 ± 1061.3 | 2388.7 ± 992.8 | 2327.9 ± 921.1 |
| Alcohol intake, g/d | 13.4 ± 27.4 | 15.0 ± 29.5 | 14.6 ± 27.7 | 14.7 ± 26.4 | 16.6 ± 32.2 |
|  |  |  |  |  |  |
| Women, n | 2,293 | 2,931 | 14,069 | 5,098 | 6,065 |
| Age, y | 57.3 ± 7.0 | 56.8 ± 7.1 | 56.0 ± 7.1 | 55.4 ± 7.1 | 54.6 ± 7.0 |
| Race/ethnicity (%) |  |  |  |  |  |
| African American | 15.9 | 15.7 | 12.3 | 9.7 | 9.7 |
| Native Hawaiian | 9.4 | 8.1 | 7.8 | 8.2 | 9.2 |
| Japanese American | 24.0 | 26.5 | 30.7 | 35.7 | 37.3 |
| Latino | 23.1 | 20.1 | 18.5 | 17.8 | 18.6 |
| White | 27.6 | 29.5 | 30.7 | 28.6 | 25.1 |
| Body weight, kg | 68.8 ± 14.8 | 67.7 ± 14.9 | 66.8 ± 14.6 | 65.6 ± 14.1 | 66.1 ± 14.5 |
| Body mass index (%) |  |  |  |  |  |
| 18.5-<25 kg/m^2^ | 44.7 | 48.0 | 51.4 | 53.6 | 51.7 |
| 25-<30 kg/m^2^ | 32.8 | 32.0 | 30.8 | 30.4 | 30.5 |
| 30-<35 kg/m^2^ | 15.4 | 13.0 | 12.0 | 11.0 | 11.9 |
| ≥35 kg/m^2^ | 7.2 | 6.9 | 5.8 | 5.0 | 5.9 |
| Education (%) |  |  |  |  |  |
| ≤High school | 38.8 | 36.6 | 32.7 | 33.0 | 33.8 |
| Vocational school/some college | 31.4 | 31.7 | 32.4 | 31.0 | 33.0 |
| ≥Graduated college | 29.8 | 31.7 | 34.9 | 36.0 | 33.2 |
| Marital status (%) |  |  |  |  |  |
| Married | 63.2 | 65.7 | 66.6 | 68.5 | 68.6 |
| Separated/divorced | 20.4 | 19.2 | 18.5 | 17.9 | 17.9 |
| Widowed | 10.1 | 9.3 | 8.7 | 7.7 | 7.5 |
| Never married | 6.2 | 5.9 | 6.1 | 5.9 | 6.0 |
| Smoking status (%) |  |  |  |  |  |
| Never | 55.6 | 57.9 | 58.0 | 58.9 | 59.2 |
| Past | 32.8 | 30.7 | 29.9 | 28.8 | 28.0 |
| Current | 11.6 | 11.5 | 12.2 | 12.3 | 12.8 |
| Physical activity^b^, h/d | 1.2 ± 1.3 | 1.3 ± 1.3 | 1.2 ± 1.3 | 1.2 ± 1.3 | 1.1 ± 1.3 |
| Multivitamin use (%) | 58.5 | 55.9 | 53.8 | 53.0 | 52.5 |
| MHT use (%) | 52.4 | 51.9 | 50.1 | 47.8 | 46.0 |
| Energy intake, kcal/d | 2222.2 ± 1051.7 | 2076.5 ± 950.8 | 1984.5 ± 892.6 | 1900.5 ± 808.4 | 1800.1 ± 742.9 |
| Alcohol intake, g/d | 4.9 ± 14.7 | 4.2 ± 11.0 | 4.8 ± 14.3 | 4.6 ± 13.6 | 4.0 ± 11.4 |

MHT, menopausal hormone therapy

^a^ Greatest decline: ≥1 SD decrease; moderate decline: 0.5–<1 SD decrease; stable: <0.5 SD change; moderate increase: 0.5–<1 SD increase; greatest increase: ≥1 SD increase ^b^ Hours in moderate vigorous activity per day
